# Microbial metabolites at the nexus of gut-brain communication and neurodevelopmental disorders

**DOI:** 10.3389/fnut.2026.1836981

**Published:** 2026-07-03

**Authors:** Yupeng Lai, Ming Zhang, Dandan Lang, Enfu Tao

**Affiliations:** 1 Department of Pediatrics, Guangdong Provincial People's Hospital, Zhuhai Hospital (Jinwan Central Hospital of Zhuhai), Zhuhai, Guangdong, China; 2 Department of Pediatrics, Zhuhai People's Hospital (Zhuhai Clinical Medical College of Jinan University), Zhuhai, Guangdong, China; 3 Department of Neonatology and NICU, Wenling Maternal and Child Health Care Hospital, Wenling, Zhejiang, China

**Keywords:** autism spectrum disorder, early-life programming, microbial metabolites, microbiota-gut-brain axis, neurodevelopmental disorders, short-chain fatty acids, Tourette syndrome

## Abstract

The microbiota-gut-brain axis (MGBA) has emerged as a critical regulator of neurodevelopment, with microbial metabolites serving as key signaling molecules that bridge the intestinal ecosystem and the central nervous system. This review gathers current evidence that connects disruptions in microbial metabolites to the pathogenesis of neurodevelopmental disorders (NDDs), including autism spectrum disorder (ASD) and attention-deficit/hyperactivity disorder (ADHD). Our comprehensive overview discusses major neuroactive metabolite classes—short-chain fatty acids (SCFAs), tryptophan derivatives, bile acids, and phenolic compounds—and their established roles functions in affecting neuroinflammation, epigenetic programming, synaptic function, and blood–brain barrier integrity. Converging evidence from human multi-omics studies and preclinical models frequently reported patterns of metabolic dysregulation in NDDs, including reduced SCFA production, altered kynurenine pathway metabolites, and accumulation of neurotoxic compounds such as para-cresol (p-cresol). However, substantial heterogeneity exists across studies, and causal evidence in humans remains predominantly associative. We further examine the critical early-life window during which the metabolite-producing microbiome is shaped by maternal factors, nutrition, and environmental exposures, with lasting consequences for neurodevelopmental trajectories. Finally, we discuss new intervention strategies such as probiotics, dietary substrates, fecal microbiota transplantation, and metabolite-based therapies, and propose a plan to transition from associative findings to causal, personalized approaches using microbial metabolites as biomarkers and therapeutic targets in child neurodevelopment.

## 1 Introduction

The human gut harbors a complex and dynamic ecosystem of trillions of microorganisms, collectively termed the gut microbiota, which plays a critical role in regulating host physiology beyond digestion (1). This microbial community is deeply integrated into host biology, functioning as a key regulator of metabolism, immune responses, and notably, brain function (1). This bidirectional communication system between gut microbes and the central nervous system (CNS) is described by the conceptual framework known as the microbiota-gut-brain axis (MGBA) (2). The MGBA represents a sophisticated network of pathways through which the gut microbiota can influence the host’s nervous system development, emotional regulation, and cognitive function (2). This axis operates via multiple interconnected channels, including immune modulation, endocrine signaling, and the production of microbial metabolites that can act directly or indirectly on neural circuits, such as those involving the vagus nerve (1). The composition and function of this gut microbial community are not static but are shaped by a confluence of factors from birth, including diet, lifestyle, genetics, and environmental exposures, evolving throughout an individual’s lifespan (1, 2).

The influence of the gut microbiota on the brain is particularly salient during critical periods of neurodevelopment. Factors such as birth method, feeding practices, and antibiotic use in early life play a significant role in shaping the gut microbiome, establishing a long-term relationship with the host that can affect brain health (1). This host-microbe dialog forms a dynamic regulatory layer over brain maturation, with significant implications for the emergence of a functional and resilient nervous system. Consequently, disruptions in the establishment or maintenance of a balanced gut microbiota during these sensitive windows have been associated with atypical neurodevelopmental trajectories (3).

Neurodevelopmental disorders (NDDs), such as autism spectrum disorder (ASD) and attention-deficit/hyperactivity disorder (ADHD), represent a heterogeneous group of conditions. A growing body of research has shifted focus toward exploring the gut microbiota as a potential driver or modulator of these conditions (2). Clinical observations frequently note co-occurring gastrointestinal symptoms in individuals with ASD, prompting investigations into whether underlying gut dysbiosis is linked to core behavioral phenotypes (4). Research has increasingly explored the potential role of gut microbiota in driving these neurodevelopmental conditions, moving beyond mere association to interrogate potential causal mechanisms (2). This trend is evident in the growing scientific literature, shifting from basic comparisons of microbial composition to exploring how gut microbes affect brain and behavior (4).

Central to this mechanistic inquiry are the diverse small molecules produced or modified by gut bacteria, collectively known as microbial metabolites. These compounds serve as primary signaling agents within the MGBA, mediating the gut’s influence on the brain. Key classes of these neuroactive metabolites include short-chain fatty acids (SCFAs) like acetate, propionate, and butyrate; derivatives of dietary tryptophan such as indoles and serotonin precursors; and various other neurotransmitters and immune-modulating molecules (2). These metabolites can enter systemic circulation, interact with the host’s immune system, stimulate enteroendocrine and vagal pathways, and, crucially, cross the blood–brain barrier (BBB) to directly affect neuroinflammation, neurotransmission, and neuronal integrity (1). The emerging hypothesis is that alterations in the production or balance of these microbial metabolites, stemming from gut dysbiosis, may disrupt normal neurodevelopmental processes and contribute to the pathophysiology of NDDs (3). Bibliometric analyses of the field confirm this focus, identifying SCFAs and the gut-brain axis as recurrent and high-impact keywords (4).

Thus, the study of the MGBA, particularly through the lens of microbial metabolites, offers a promising framework for understanding the complex etiology of NDDs. It bridges environmental influences with biological pathways affecting brain development and function. This introduction outlines the role of microbial metabolites in the gut-brain axis, their disruption in conditions like ASD and ADHD, and the mechanisms connecting these metabolites to brain function and behavior. Ultimately, this knowledge base informs the development of novel microbiome-targeted intervention strategies, which aim to restore a healthy microbial metabolic output and thereby mitigate neurodevelopmental dysfunctions (2, 3).

## 2 Microbial metabolites as key communicators: an overview of SCFAs, tryptophan derivatives, and other neuroactive compounds

Building upon the foundational concept of the MGBA, it becomes clear that microbial metabolites serve as the primary biochemical language through which the gut microbiota communicates with the host, including the developing brain. These small molecules, produced or modified by intestinal bacteria from dietary substrates, can enter systemic circulation, cross or signal across biological barriers, and directly or indirectly modulate peripheral and CNS physiology. Their influence spans immune regulation, epithelial barrier integrity, endocrine signaling, and neural function, positioning them as critical mediators in the context of neurodevelopment. This section summarizes key classes of microbially-derived metabolites involved in brain communication, highlighting SCFAs, tryptophan derivatives, and other emerging neuroactive compounds, supported by recent human and preclinical studies.

SCFAs, including acetate, propionate, and butyrate, are produced primarily by the bacterial fermentation of dietary fiber in the colon. Beyond their local roles in gut health, SCFAs have systemic immunomodulatory and neuroactive properties. Evidence suggests their protective role begins early in life. For instance, in a human cohort study, infants who later developed sensitization, atopic eczema, or food allergy were found to have significantly lower plasma concentrations of several SCFAs, such as formic, acetic, succinic, and caproic acid, at 4 months of age (5). A systematic review supports this, indicating that the three main SCFAs in early childhood have a protective effect against the development of allergic diseases like atopic dermatitis and asthma (6). These findings point to the early-life immunoregulatory potential of SCFAs, which is pertinent given the common comorbidity of atopy and specific NDDs. The source of early-life SCFAs is crucial; human milk contains a rich profile of SCFAs, with butyric and caproic acid appearing to be actively enriched, suggesting a potential delivery mechanism from mother to infant (5, 7). Importantly, maternal SCFA status can directly impact offspring development. In a model of gestational diabetes, reduced maternal SCFAs impaired G-protein coupled receptor (GPR) 43 signaling in the fetus, leading to congenital anomalies of the kidney and urinary tract, highlighting a direct link from maternal gut metabolism to fetal organogenesis (8).

Butyrate, in particular, has garnered significant attention for its potent anti-inflammatory and epigenetic-modifying functions, primarily through inhibition of histone deacetylases (HDACs). Its therapeutic potential extends to neurodevelopmental and neuroinflammatory models. In a rat model of Tourette syndrome (TS), the traditional medicine Jing An decoction was shown to alleviate symptoms by enriching butyrate-producing bacteria (Lachnospiraceae NK4A136 group), elevating butyrate levels in the colon and striatum, and subsequently suppressing neuroinflammation via inhibition of the HDAC3/Toll-like receptor 4 (TLR4)/nuclear factor kappa-light-chain-enhancer of activated B cells (NF-κB) pathway in microglia (9). Similarly, butyrate rescued chlorpyrifos-induced social deficits in zebrafish, an effect mediated through the inhibition of class I HDACs, which reversed the pesticide-induced suppression of neuronal gene expression (10). Beyond behavior, butyrate exhibits protective effects on neurovascular development, as demonstrated by its capacity to protect against pathological angiogenesis and neuronal damage in preclinical models of retinopathy of prematurity (11). These studies collectively illustrate the multi-faceted role of butyrate in supporting brain health by modulating inflammation, gene expression, and cellular function.

The metabolism of the essential amino acid tryptophan represents another major communication pathway within the MGBA. Gut microbiota significantly influence the host’s tryptophan metabolism, diverting it into various pathways that yield metabolites with profound neuroactive effects. These include the serotonin pathway, the microbial indole pathway, and the kynurenine pathway. Alterations in these metabolites are strongly linked to neurodevelopmental and neurological outcomes. A pivotal cross-sectional study in children with ASD found that fecal levels of specific tryptophan-related metabolites, including kynurenate, were significantly lower compared to neurotypical controls (12). These metabolites were not only correlated with ASD severity and sensory sensitivities but were also associated with altered brain activity in the insula and cingulate cortex—regions critical for interoception and emotion processing. Furthermore, brain activity in these regions mediated the relationship between gut metabolites like indolelactate and clinical symptoms, providing a direct link between gut-derived molecules, brain function, and behavior (12).

Individual tryptophan metabolites show distinct mechanistic actions. Indole-3-propionic acid (IPA), a tryptophan-derived microbial metabolite, demonstrated therapeutic potential in a 16p11.2 microdeletion mouse model of ASD. These mice exhibited gut dysbiosis, reduced IPA levels, and social and cognitive deficits. Oral administration of IPA ameliorated these behavioral impairments and restored hippocampal inhibitory synaptic transmission by activating the ERK1 signaling pathway (13). This highlights a scenario where a genetic neurodevelopmental risk factor converges with a microbial metabolic deficit, and supplementation of the metabolite can rescue phenotypes. On the other hand, the kynurenine pathway metabolite kynurenic acid (KYNA) has been implicated in neuropsychiatric disorders. While elevated KYNA is linked to schizophrenia and associated with excessive microglia-mediated synapse elimination (14), reduced levels, as seen in the ASD study (12), may indicate a different dysregulation. Interestingly, in pediatric migraine, a significant decrease in plasma KYNA was observed alongside an increase in the neurotoxic metabolite quinolinic acid, and the KYNA/quinolinic acid ratio showed excellent diagnostic efficacy (15). This demonstrates how the balance of tryptophan pathway metabolites, influenced by gut microbes, can be diagnostically and mechanistically relevant across different neurological and neurodevelopmental conditions.

Beyond SCFAs and tryptophan derivatives, other classes of microbial metabolites are emerging as key players in host physiology and neurodevelopment. Bile acids, classically known for lipid digestion, are extensively modified by the gut microbiota into secondary bile acids and, as recently discovered, a diverse array of microbially conjugated bile acids (MCBAs). These molecules act as potent signaling molecules through receptors such as farnesoid X receptor (FXR) and Takeda G protein-coupled receptor 5 (TGR5). Furthermore, bile acid profiles are altered in other immune-related conditions; children with the combined asthma and atopy phenotype (At^+^As^+^) showed higher plasma levels of bile acids (16), and specific fecal bile acids predicted the success or failure of peanut oral immunotherapy, linking microbial bile acid metabolism to allergic tolerance (17, 18).

Other notable metabolites include trimethylamine N-oxide (TMAO) and phenolic compounds like p-cresol. TMAO, derived from microbial metabolism of dietary choline and carnitine, is implicated in cardiovascular and metabolic diseases. In pregnancy, elevated TMAO levels are associated with preeclampsia, and its inhibition in mice improves pregnancy outcomes by reducing oxidative stress and inflammation (19). While not directly neurodevelopmental, this underscores how a microbial metabolite can impact the *in utero* environment, with potential consequences for fetal brain development. More directly neurotoxic is p-cresol, a phenolic compound produced by certain gut bacteria. In human studies, elevated urinary levels of its sulfate conjugate, p-cresol sulfate, are a recurrent finding in a subset of children with ASD (20). Mechanistically, using mouse models, p-cresol and p-cresol sulfate have been shown to inhibit the host enzymes tyrosine hydroxylase and dopamine-β-hydroxylase (DBH), critical for catecholamine synthesis. This inhibition reduces dopamine neuron excitability in the ventral tegmental area and is sufficient to induce social deficits in mice, providing a direct molecular pathway linking a gut bacterial metabolite to ASD-relevant behaviors (21).

The diversity of microbial metabolites and their mechanisms is further exemplified by bioactive components derived from specific bacteria, such as the outer membrane protein Amuc_1100 from *Akkermansia muciniphila*. This protein, which can signal through Toll-like receptor 2, has been shown to attenuate cardiovascular inflammation in a model of Kawasaki disease vasculitis, an effect also achieved by supplementing SCFAs (22). This illustrates that microbial communication extends beyond small molecules to include bacterial structural components, all contributing to the regulation of systemic and potentially neuroinflammatory states (23).

**Figure 1:**
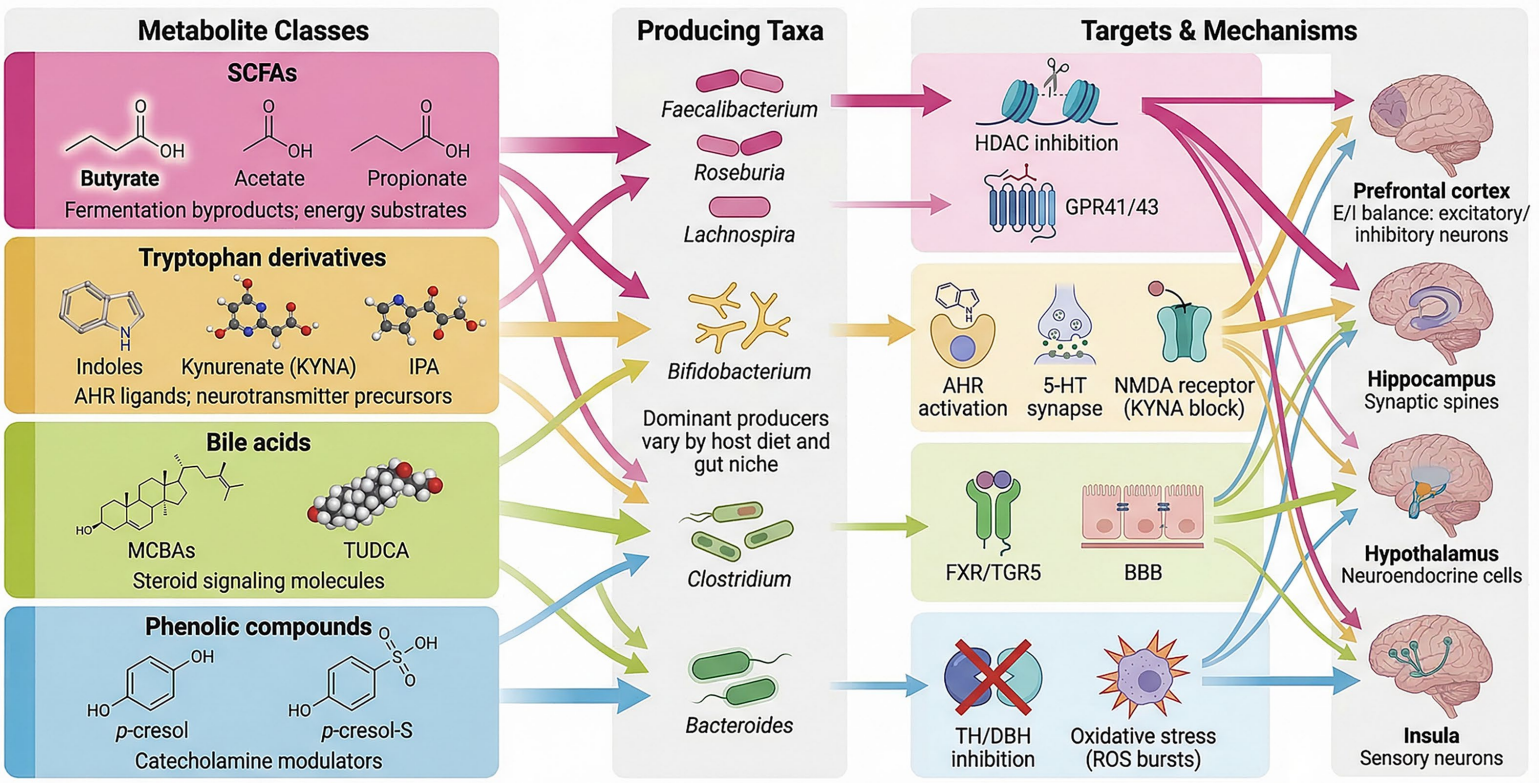
Gut microbial metabolites as key signaling molecules along the brain-gut axis. Schematic overview of the major classes of microbiota-derived metabolites implicated in gut-brain communication, their producing bacterial taxa, and their molecular targets and neurobiological effects. (Left) Four principal classes of neuroactive microbial metabolites are depicted: short-chain fatty acids (SCFAs) (butyrate, acetate, propionate), tryptophan derivatives [indoles, kynurenate, indole-3-propionic acid (IPA)], bile acids [microbially-conjugated bile acids (MCBAs), tauroursodeoxycholic acid (TUDCA)], and phenolic compounds (p-cresol, p-cresol sulfate). (Center) Key bacterial genera responsible for the production of each metabolite class, including *Faecalibacterium*, *Roseburia*, and *Lachnospiraceae* for SCFAs; *Bifidobacterium*, *Clostridium*, and *Bacteroides* for tryptophan metabolites and bile acids; and *Clostridium* and Bifidobacterium for phenolic compounds. (Right) Molecular mechanisms and neural targets through which these metabolites exert their effects. SCFAs modulate host physiology via histone deacetylase (HDAC) inhibition (particularly butyrate), G-protein-coupled receptor (GPR41/43) activation, and energy homeostasis. Tryptophan derivatives activate the aryl hydrocarbon receptor (AHR), regulate serotonin (5-HT) synthesis, and modulate NMDA receptor activity via kynurenic acid (KYNA). Bile acids signal through farnesoid X receptor (FXR) and Takeda G-protein-coupled receptor 5 (TGR5), influence blood–brain barrier integrity, and regulate neuroendocrine function. Phenolic compounds, notably p-cresol, inhibit catecholamine biosynthesizing enzymes [tyrosine hydroxylase (TH), dopamine-β-hydroxylase (DBH)] and induce oxidative stress. These metabolite signals converge on key brain regions implicated in neurodevelopment and behavior, including the prefrontal cortex (excitatory/inhibitory balance), hippocampus (synaptic plasticity), hypothalamus (neuroendocrine control), and insula (interoception and sensory integration). Arrow thickness reflects the relative strength of evidence for each pathway. SCFAs, short-chain fatty acids; IPA, indole-3-propionic acid; MCBA, microbially-conjugated bile acid; TUDCA, tauroursodeoxycholic acid; HDAC, histone deacetylase; GPCR, G-protein-coupled receptor; AHR, aryl hydrocarbon receptor; 5-HT, serotonin; KYNA, kynurenic acid; NMDA, N-methyl-D-aspartate; FXR, farnesoid X receptor; TGR5, Takeda G-protein-coupled receptor 5; TH, tyrosine hydroxylase; DBH, dopamine-β-hydroxylase; BBB, blood–brain barrier; PFC, prefrontal cortex; E/I, excitatory/inhibitory. Created with BioRender.com.

In summary, the gut microbiota produces a vast repertoire of metabolites—SCFAs, tryptophan derivatives, bile acids, phenolics, and others—that serve as essential communicators along the gut-brain axis. These molecules influence host physiology through receptors, enzyme inhibition, epigenetic modification, and immune modulation. As the subsequent sections will detail, disruptions in the production or balance of these key microbial metabolites are increasingly documented in specific NDDs, providing plausible biological pathways through which the gut microbiome may contribute to the pathogenesis of conditions like ASD, ADHD, and TS. Figure 1 provides a visual summary of these diverse metabolite classes, their bacterial origins, and the molecular pathways through which they influence brain development and function.

## 3 Disrupted microbial metabolites in specific neurodevelopmental disorders: evidence from ASD, ADHD, and related conditions

Building upon the fundamental roles of microbial metabolites as gut-brain communicators, converging evidence from human observational studies, multi-omics analyses, and experimental models highlights distinct disruptions in these chemical signatures within specific neurodevelopmental conditions. The most extensive body of evidence concerns ASD, where alterations span multiple metabolic pathways. Notably, substantial heterogeneity persists across studies due to differences in age, diet, medication use, and analytical platforms. A systematic review of human observational studies has consolidated findings of altered SCFAs and neurotransmitter levels in ASD (24). Specifically, in a cross-sectional metabolomic study of children, fecal metabolomic profiling reveals significantly lower levels of tryptophan-related metabolites, including kynurenate, in children with ASD compared to neurotypical controls (12). These reduced levels correlate not only with core ASD severity scores but also with altered neural activity in the insular and cingulate cortices—brain regions critical for interoception and socio-emotional processing (12). Furthermore, a specific subset of microbial tryptophan metabolites, namely indolelactate and tryptophan betaine, shows a mediated relationship with ASD symptom severity through their association with activity in these same brain regions (12). Beyond tryptophan, the phenylalanine-derived microbial metabolite p-cresol and its host-conjugated form, p-cresol sulfate, are frequently elevated in ASD (20, 21). Experimental evidence in mice have shown that p-cresol impairs social behavior. This metabolite inhibits two key enzymes involved in catecholamine production—tyrosine hydroxylase (TH) and DBH, within brainstem regions. These findings establish a direct connection between a gut-derived compound and dysfunction in reward circuitry and social behavior (21). A multi-site investigation has identified that many children with ASD show increased urinary levels of various microbially-derived metabolites (MDMs), particularly those originating from phenylalanine and tryptophan metabolism. These findings suggest that this metabolic signature may define a distinct ASD sub-phenotype, as it demonstrated high diagnostic sensitivity and specificity (20).

The gut microbial ecosystem’s broader functional state in ASD extends beyond bacteria to include the virome. Metagenomic analysis indicates an altered gut virome composition in children with ASD, characterized by enrichment of specific bacteriophages (25). Crucially, the disrupted interplay between the gut bacteriome and virome appears to influence the microbial genomic capacity for synthesizing neuroactive metabolites, suggesting a complex ecological disturbance underpinning metabolic imbalances (25). Interventions that modulate the microbiota and its metabolic output support the pathogenic relevance of these findings. Probiotic administration, such as *Limosilactobacillus reuteri* or *Lacticaseibacillus rhamnosus* GG, ameliorates social deficits in rodent ASD models by modifying SCFA profiles (increasing butyrate, decreasing propionate), enhancing gut barrier function, and reducing systemic inflammation and HPA axis activation (26). Similarly, the probiotic *Bifidobacterium adolescentis* DM8504 alleviates autistic-like behaviors in a valproic acid rat model, concurrently restoring fecal SCFA levels and enriching SCFA-producing bacterial taxa (27). In mice, dietary supplementation with L-tyrosine reduces ASD-like behaviors. This beneficial effect can be transferred to other mice through fecal microbiota transplantation (FMT). The underlying mechanisms involve remodeling of gut microbiota and changes in neurotransmitter profiles within hippocampal (28). FMT itself, particularly from donors with high *Lactobacillus* abundance, improves social interaction in a propionic acid-induced mouse model of ASD, restoring gut microbiota diversity, normalizing prefrontal cortex excitatory/inhibitory balance, and reducing propionic acid levels in the brain (29). Even non-digestible oligosaccharides like 2′-Fucosyllactose show efficacy, improving ASD-like behaviors in a maternal immune activation model by modulating gut microbiota (increasing *Akkermansia* and *Bifidobacterium*) and elevating brain bile acid levels (30).

Although the evidence base for ADHD is less extensive than for ASD, several recent studies have reported promising findings that merit discussion. Parallel research has implicated microbial metabolite disturbances in ADHD. A systematic review notes gut microbiota alterations in ADHD, including correlations between specific bacterial genera (*Faecalibacterium*, *Bacteroides*) and hyperactivity/impulsivity (24). More recent, in-depth analyses reveal symptom-specific microbial and metabolic profiles. In a human study, shotgun metagenomic and metabolomic studies in ADHD patients identify a deficiency in SCFA synthesis as a key feature, with the beneficial bacterium *Fructilactobacillus sanfranciscensis* notably downregulated and linked to all core ADHD symptoms (inattention, hyperactivity, impulsivity) (31). This study established a causal mediation link where imidazoleacetic acid partially mediated the effects of reduced *Fructilactobacillus sanfranciscensis* on inattention, and supplementation with acetate alleviated inattention in a mouse model receiving fecal transplants from ADHD patients (31). Other functional alterations include a lower abundance of gut bacterial gene modules responsible for vitamin B12 biosynthesis, particularly observed in children with ADHD on psychostimulant medication, which was associated with looser stool consistency (32). This suggests medication and gut transit time may interact with microbial metabolic function. Synbiotic treatment in ADHD patients can modulate the gut microbiome and increase plasma SCFA levels toward normal, indicating the modifiability of these pathways (32). The involvement of tryptophan metabolism is further suggested by a prospective birth cohort study finding that higher levels of *Bifidobacterium* in the one-week neonatal gut microbiome, and its derived metabolite indole-3-lactic acid (ILA) in neonatal blood, were associated with an increased risk of ADHD at age 10 (33). This points to a critical, and potentially adverse, role of very early microbial metabolic programming in neurodevelopmental trajectories. Traditional interventions also appear to act through metabolite modulation; Xiaoer Huanglong Pellets improve ADHD-like behaviors in rats by restoring gut microbial homeostasis and correcting abnormalities in amino acid metabolism, neurotransmitter regulation, and SCFA production (34).

Evidence for microbial metabolite disruption extends to other neurodevelopmental and neuropsychiatric conditions. In TS, the herbal formulation Jing An decoction alleviates symptoms in a rat model by increasing the abundance of butyrate-producing bacteria (e.g., *Lachnospiraceae* NK4A136 group) and elevating butyrate levels in the colon and striatum (9). The elevated butyrate subsequently reduces neuroinflammation by inhibiting the TLR4/HDAC3/NF-κB pathway and promoting anti-inflammatory M2 microglial polarization (9). In pediatric migraine, another condition often comorbid with NDDs, alterations in gut microbiota composition are accompanied by significant changes in plasma tryptophan metabolites, including decreased kynurenic acid and increased serotonin and quinolinic acid, with the kynurenic/quinolinic acid ratio showing excellent diagnostic potential (15). This suggests shared mechanisms of gut-brain communication via tryptophan pathways across different brain-based disorders. Furthermore, Tuberous Sclerosis Complex (TSC) is frequently associated with epilepsy and neurodevelopmental issues. A pilot study investigating this condition revealed gut microbiota alterations similar to those foundin an epilepsy cohort. These included a decrease in Firmicutes and a notable enrichment of *Akkermansiaceae*—a bacterial genus also observed in other NDDs (35).

The impact of environmental exposures on this vulnerable gut-brain-metabolite axis is increasingly recognized. Prenatal exposure to certain per- and polyfluoroalkyl substances (PFAS), specifically PFHxS, is associated with poorer neurobehavioral outcomes in childhood. This association appears to involve changes in the gut microbiota. Specifically, researchers observed reduced microbial diversity, and lower relative abundances of SCFA-producing genera like *Ruminococcus gauvreauii* group, with gut microbiota alpha diversity acting as a mediator (36). Maternal immune status itself can program offspring metabolism, as maternal overexposure to the bacterial peptidoglycan motif muramyl dipeptide (MDP) during late gestation alters neurodevelopment and social behavior in juvenile offspring in a sex-specific manner, independent of overt inflammation (37). Even pharmacological treatments for neurodevelopmental symptoms can have off-target effects on this axis. Early-life exposure to the antipsychotic risperidone impairs cognitive function in mice, an effect transferable via FMT and linked to gut microbiota dysbiosis, a reduction in the neuroprotective bile acid tauroursodeoxycholic acid (TUDCA), and accumulation of the microbial tyrosine metabolite p-cresol in the brain (38).

**Figure 2:**
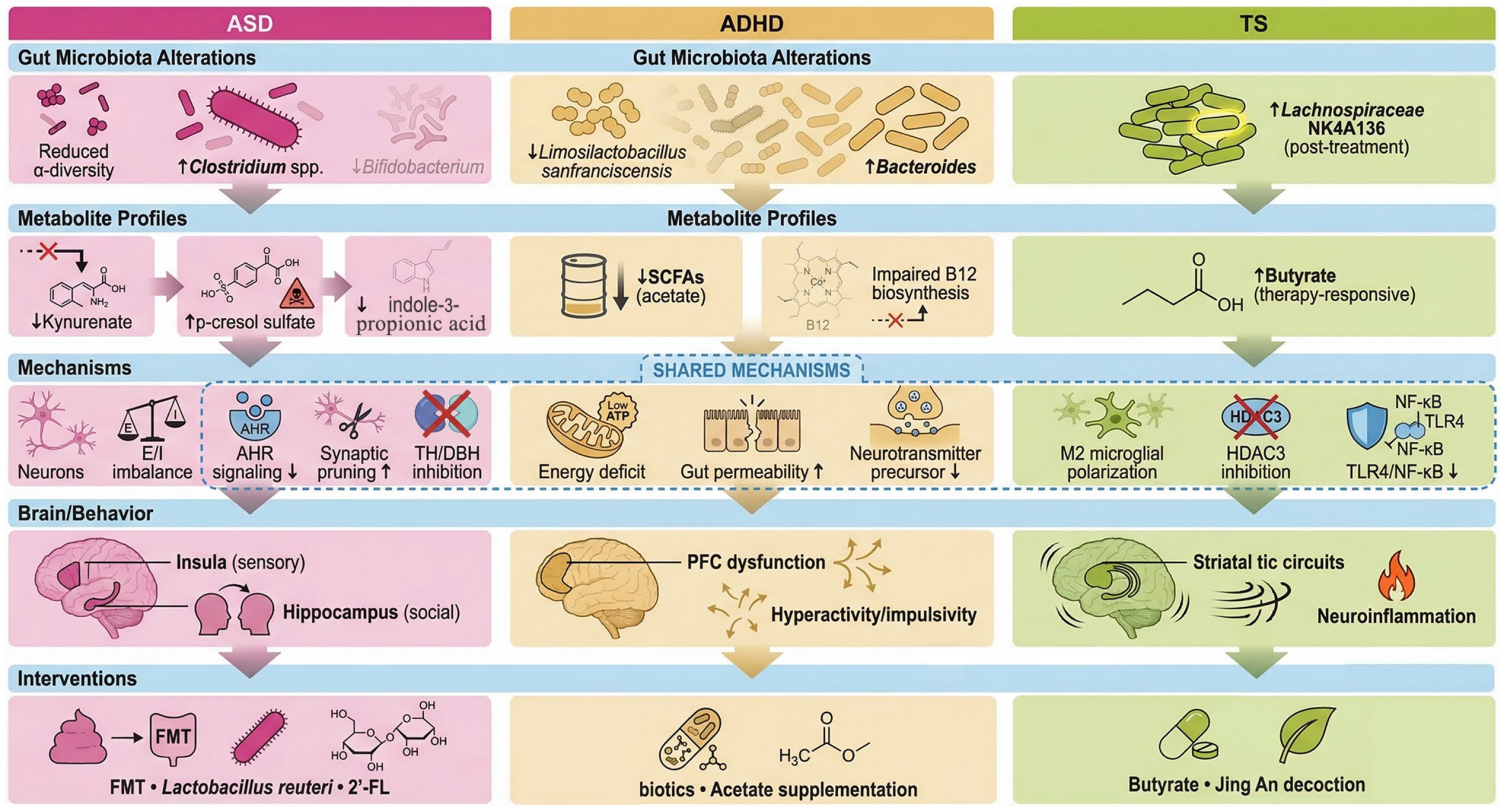
From gut dysbiosis to neural dysfunction: metabolic signatures in neurodevelopmental disorders. Integrated multi-level comparison of gut microbial and metabolic alterations, underlying mechanisms, brain correlates, and intervention evidence across three major neurodevelopmental disorders: autism spectrum disorder (ASD), attention-deficit/hyperactivity disorder (ADHD), and Tourette syndrome (TS). Each disorder is represented in a separate column, with rows progressing from proximal (gut) to distal (brain and behavior) levels of analysis. (First row) Gut microbiota alterations. ASD is characterized by reduced microbial diversity, enrichment of *Clostridium species*, and depletion of *Bifidobacterium*. ADHD shows a specific reduction in *Limosilactobacillus sanfranciscensis* and increased *Bacteroides*. TS is associated with increased *Lachnospiraceae NK4A136* group following effective treatment. (Second row) Microbial metabolite profiles. ASD features decreased kynurenate, indolelactate, and indole-3-propionic acid (IPA), alongside elevated p-cresol and p-cresol sulfate. ADHD shows a consistent deficiency in short-chain fatty acids (SCFAs), particularly acetate, and reduced vitamin B12 biosynthesis pathway abundance. TS is marked by increased butyrate levels in responders to treatment. (Third row) Mechanistic pathways. In ASD, p-cresol inhibits tyrosine hydroxylase (TH) and dopamine-β-hydroxylase (DBH), reduced indole metabolites impair aryl hydrocarbon receptor (AHR) signaling, and kynurenate deficiency may alter synaptic pruning. Altered excitatory/inhibitory (E/I) balance is a convergent mechanism. In ADHD, SCFA deficiency impairs energy metabolism and neurotransmitter precursor availability, with associated gut barrier dysfunction. In TS, butyrate inhibits HDAC3 and suppresses TLR4/NF-κB signaling, promoting anti-inflammatory M2 microglial polarization. (Fourth row) Brain regions and behavioral correlates. ASD-associated metabolite changes correlate with altered activity in the insula and anterior cingulate cortex (sensory integration and interoception) and hippocampus (social behavior). ADHD metabolite profiles link to prefrontal cortex dysfunction underlying inattention and hyperactivity/impulsivity. TS involves striatal circuitry mediating tics, with neuroinflammation as a key feature. (Fifth row) Intervention evidence. In ASD, fecal microbiota transplantation (FMT), *Lactobacillus reuteri*, 2′-fucosyllactose (2′-FL), and IPA supplementation show preclinical efficacy. In ADHD, synbiotic therapy and acetate supplementation ameliorate symptoms in models. In TS, the herbal formulation Jing An decoction and butyrate supplementation alleviate tics and neuroinflammation. ASD, autism spectrum disorder; ADHD, attention-deficit/hyperactivity disorder; TS, Tourette syndrome; SCFAs, short-chain fatty acids; IPA, indole-3-propionic acid; TH, tyrosine hydroxylase; DBH, dopamine-β-hydroxylase; AHR, aryl hydrocarbon receptor; KYNA, kynurenic acid; HDAC, histone deacetylase; TLR4, Toll-like receptor 4; NF-κB, nuclear factor kappa-light-chain-enhancer of activated B cells; E/I, excitatory/inhibitory; PFC, prefrontal cortex; ACC, anterior cingulate cortex; FMT, fecal microbiota transplantation; 2′-FL, 2′-fucosyllactose. Created with BioRender.com.

Importantly, these metabolic disturbances are not isolated events but often reflect a fundamental breakdown in the functional ecology of the gut microbiome. As noted in conditions like ASD, changes in the bacteriome-virome interplay can compromise the community’s capacity to produce beneficial neuroactive metabolites (25). This systems-level perspective suggests that NDDs may be characterized not merely by the absence or presence of single microbial species, but by a dysregulated metabolic network emanating from the gut. The convergence of findings—spanning SCFA deficiency in ADHD (31), tryptophan pathway alterations in ASD (12), bile acid changes in TS (9) and ASD models (30), and the pathological rise of phenolic compounds like p-cresol in ASD (20, 21)—paints a picture of a multifaceted metabolic disequilibrium. This disequilibrium is sensitive to early-life influences (33, 36, 37), is modifiable by dietary, probiotic, and microbial interventions (26, 27, 29, 32, 34), and is mechanistically linked to core neuropathological processes such as neuroinflammation, excitatory/inhibitory imbalance, and synaptic dysfunction (9, 29, 38). Figure 2 integrates these multi-level alterations across ASD, ADHD, and TS, providing a comparative framework that links gut microbial and metabolic disturbances to mechanistic pathways, brain correlates, and emerging intervention evidence. Table 1 provides a systematic overview of the key microbial metabolite alterations, associated bacterial taxa, proposed mechanisms, and intervention evidence across these NDDs, complementing the visual framework in Figure 2.

**Table 1:** Microbial metabolite alterations in major neurodevelopmental disorders.

Disorder	Key metabolite changes	Associated bacterial taxa	Proposed mechanisms	Evidence sources	Intervention evidence
Autism Spectrum Disorder (ASD)	↓ Tryptophan derivatives Kynurenate, Indolelactate, IPA ↑ Phenolic compounds p-Cresol and sulfate Altered SCFAs ↓ Butyrate, Acetate ↑ Propionate, Valeric acid	↑ *Clostridium* spp. (propionate producers) ↓ *Bifidobacterium* spp. ↑ *Bacteroides* (subset) ↓ *Lachnospiraceae* (butyrate producers) (24)	Catecholamine synthesis inhibition (p-cresol via TH/DBH) (21) Altered synaptic pruning/function (↓ KYNA) (12). E/I imbalance (↓ GABAergic tone) (29) • Gut barrier and immune dysfunction (26) • Impaired AHR signaling (↓ indoles) (63)	Human: Fecal metabolomics (12); Urinary MDM (20) Preclinical: Animal models (13, 21, 26, 29)	Preclinical: FMT (29) Probiotics (*L. reuteri*, *L. rhamnosus* GG) (26) *B. adolescentis* DM8504 (27) 2′-FL (30) IPA (13) L-tyrosine (28) *L. fermentum* K73 synbiotic (65, 66)
Attention-Deficit/Hyperactivity Disorder (ADHD)	↓ SCFAs (Acetate, Butyrate, Propionate) ↓ Vitamin B12 biosynthesis Bifidobacterium-ILA axis ↑ Neonatal ILA (risk factor)	↓ *Limosilactobacillus sanfranciscensis* (31) ↑*Bacteroides* (symptom-specific) (24) ↑ *Bifidobacterium* (neonatal, risk-associated) (33)	Energy metabolism and neurotransmitter precursor deficiency (31) Gut-brain signaling via imidazoleacetic acid (partial mediator) (31) Dysregulated tyrosine/dopamine metabolism (31, 38) Medication-microbiome interactions (32)	Human: Metagenomics and metabolomics (31, 32); Birth cohort (neonatal ILA) (33); Systematic review (includes ADHD) (24) Preclinical: Animal model (31)	Clinical: Synbiotic 2000 → ↑ plasma SCFAs (32) Preclinical: Acetate supplementation (31) Xiaoer Huanglong Pellets (34)
Tourette Syndrome (TS)	↑ Butyrate (post-treatment effect)	↑ *Lachnospiraceae* NK4A136 group (butyrate producer) (9)	HDAC3 inhibition and epigenetic modulation (9) TLR4/NF-κB suppression and reduced neuroinflammation (9) M2 microglial polarization (9)	Preclinical: Rat model (Jing An decoction) (9)	Preclinical: Jing An decoction (9) Butyrate (mechanistically implicated) (9)
Pediatric migraine	↓ Kynurenic acid (KYNA) ↑ Quinolinic acid ↓ KYNA/Quinolinic acid ratio	↓ *Akkermansia*, ↓ *Faecalibacterium* ↑ *Bacteroides* (15)	Neurotoxic/neuroprotective imbalance (NMDA receptor dysregulation) (15)	Human: Plasma metabolomics and 16S sequencing (15)	Diagnostic biomarker: KYNA/Quinolinic acid ratio (high diagnostic AUC) (15)
Tuberous Sclerosis Complex (TSC)	Not yet characterized	↓ Firmicutes (e.g., *Ruminococcaceae*) ↑ *Akkermansiaceae* (35)	Shared pathways with epilepsy (hypothesized neuroinflammation) (35)	Human: Pilot case–control study (35)	None reported

Arrow directions (↑/↓) indicate direction of change in affected individuals compared to neurotypical controls unless otherwise specified. SCFAs, short-chain fatty acids; IPA, indole-3-propionic acid; ILA, indole-3-lactic acid; TH, tyrosine hydroxylase; DBH, dopamine-β-hydroxylase; AHR, aryl hydrocarbon receptor; KYNA, kynurenic acid; E/I, excitatory/inhibitory; HDAC, histone deacetylase; TLR4, Toll-like receptor 4; NF-κB, nuclear factor kappa-light-chain-enhancer of activated B cells; FMT, fecal microbiota transplantation; 2’-FL, 2′-fucosyllactose; MDM, microbially-derived metabolite; NMDA, N-methyl-D-aspartate; rRNA, ribosomal RNA.

## 4 Mechanistic pathways: from gut-derived molecules to brain function and behavior

Before detailing the molecular pathways, it is important to clarify the nature of the evidence cited in this section. Findings derived from animal models (rodents, zebrafish) and *in vitro* systems establish mechanistic plausibility under controlled conditions. In contrast, evidence from human observational studies—unless explicitly noted as interventional—remains predominantly correlational. Throughout this section, we have aimed to clearly indicate the model system or study design for each cited finding to help readers assess the strength of the evidence base.

The multifaceted metabolic disequilibrium observed in NDDs is not a terminal event but the origin of a complex cascade of biological signals. This cascade traverses the gut-brain axis, where microbial metabolites function as key physiological messengers, modulating host physiology through a repertoire of established and emerging molecular pathways. These gut-derived molecules influence brain development and function by engaging peripheral organs, crossing or modulating barrier systems, and directly interacting with neuronal and glial cells to alter neurochemistry, synaptic plasticity, and circuit dynamics.

The journey of these metabolites begins with their entry into systemic circulation. Specific transporters facilitate the absorption of microbial products from the intestinal lumen. For instance, bacterial peptidoglycan motifs like MDP can be transported across the epithelium by solute carrier transporters such as Slc15a1/PepT1 and Slc15a2/PepT2, expressions of which are upregulated in fetal brains following maternal MDP exposure (37). Similarly, conjugated metabolites like p-cresol sulfate, derived from the microbial metabolite p-cresol, accumulate in both peripheral and central matrices (21). Once in circulation, these molecules face the critical gatekeeping function of the BBB. Integrity of the BBB is itself modulated by the gut microbial milieu. Interventions that restore microbial balance, such as the traditional Chinese medicine formulation Jing An decoction or Xiaoer Huanglong Pellets, have been shown to enhance the expression of tight junction proteins, thereby repairing compromised intestinal and BBB integrity (9, 34). This suggests that a dysbiotic gut can contribute to a “leaky” gut-brain axis, potentially allowing inappropriate passage of metabolites or inflammatory mediators into the CNS.

Among the most potent gut-brain communicators are SCFAs like butyrate, acetate, and propionate. Their mechanisms are pleiotropic, spanning epigenetic regulation, immune modulation, and energy metabolism. Butyrate acts as an inhibitor of histone deacetylase (HDAC), giving it the ability directly alter gene expression within the brain. In zebrafish exposed to the pesticide chlorpyrifos, treatment with butyrate reversed resulting social deficits. This rescue effect occurred through inhibiting of class I HDACs, particularly HDAC1, which restored the expression of suppressed by CPF—including many genes linked to ASD risk (10). This epigenetic action is complemented by potent anti-inflammatory effects. In a TS rat model, the therapeutic effect of Jing An decoction was linked to increased production of butyrate, which inhibited the TLR4/HDAC3/NF-κB signaling pathway in microglia, promoting a protective M2 polarization state and alleviating neuroinflammation (9). Butyrate’s benefits extend to mitigating oxidative stress, which can damage barrier function; it protects intestinal epithelial cells by suppressing reactive oxygen species (ROS)-mediated Notch signaling activation (39). Importantly, SCFA signaling is also mediated by specific G-protein coupled receptors (GPCRs) such as GPR43. Maternal deficiency in SCFAs during gestational diabetes was shown to impair GPR43 signaling in the fetus, leading to congenital kidney abnormalities, highlighting the role of these metabolites and their receptors in guiding organogenesis, including likely aspects of brain development (8).

Tryptophan-derived metabolites act through another set of sophisticated mechanisms, directly interfacing with neurotransmission and neuroimmune interactions. The KYNA is implicated in schizophrenia pathology. In patient-derived cellular models, KYNA was found to promote microglia-mediated engulfment of synaptic structures by reducing neuronal activity, linking elevated KYNA levels to the excessive synaptic pruning characteristic of the disorder (14). Another branch of tryptophan metabolism yields indole derivatives. Indole-3-propionic acid (IPA) demonstrated therapeutic potential in a 16p11.2 microdeletion mouse model of ASD. The social and cognitive deficits in these mice, associated with reduced hippocampal inhibitory synaptic transmission, were rescued by IPA supplementation. Mechanistically, IPA activated extracellular signal-regulated kinase 1 (ERK1), a protein encoded within the 16p11.2 region, thereby restoring synaptic and behavioral phenotypes (13). Indole metabolites also serve as ligands for the aryl hydrocarbon receptor (AHR), a transcription factor crucial for intestinal and immune homeostasis. Host genetics can influence how the body responds to microbial metabolites. For example, genetic variations that affect AHR activity—including the inflammatory bowel disease (IBD)-risk locus rs1077773—modulate immune cell cytokine production. This host-microbial metabolite interaction helps shape inflammatory tone, a factor that plays an important role in neurodevelopment (40). Furthermore, microbial metabolism of tryptophan competes with the host’s serotonin synthesis pathway. Specific tryptophan metabolites like indolelactate and tryptophan betaine have been associated in human studies with altered activity in the insula and cingulate cortex. These brain regions play key roles in interoception and emotion processing. Notably, this brain activity was found to mediate the connection between those gut metabolites and the severity of ASD symptom (12).

Bile acids, classically known for lipid digestion, have emerged as potent signaling molecules with neuroactive potential. Secondary bile acids, such as TUDCA, can exhibit neuroprotective properties. In a model of risperidone-induced cognitive impairment, the observed reduction in brain TUDCA levels contributed to neuronal endoplasmic reticulum (ER) stress, while its restoration prevented cognitive deficits (38). Beyond the gut, bile acids signal through receptors like TGR5. Muricholic acid (MCA), elevated in girls with central precocious puberty, was shown to activate the TGR5-PI3K/Akt–mTOR pathway in gonadotropin-releasing hormone (GnRH) neurons, thereby promoting puberty initiation (41). This reveals a direct link between a circulating microbiome-influenced metabolite and the neuroendocrine system governing development.

Some microbial metabolites exert their effects through direct biochemical interference with host enzymes. A prime example is p-cresol, a phenolic compound frequently elevated in ASD. p-cresol and its sulfate conjugate accumulate in the brainstem and act as direct inhibitors of TH and DBH, two key enzymes in catecholamine biosynthesis. This inhibition reduces dopamine and norepinephrine production, which mechanistically explains the observed social deficits and reduced excitability of ventral tegmental area dopamine neurons in p-cresol-exposed mice (21). This represents a clear pathway where a gut bacterial product directly modulates host brain neurochemistry by inhibiting enzymatic activity.

The final common pathways through which these diverse signals converge to influence behavior often involve core neuropathological processes: neuroinflammation, excitatory/inhibitory (E/I) imbalance, and synaptic dysfunction. Neuroinflammation, frequently driven by microglial activation, is a recurrent theme. Butyrate (9), probiotics like *Lactobacillus reuteri* (26), and FMT (42) can all suppress neuroinflammatory pathways (e.g., NLRP3, NF-κB). E/I balance is critically targeted, as seen in a propionic acid-induced ASD model where targeted FMT normalized the prefrontal cortex glutamate/gamma-aminobutyric acid (GABA) ratio and corrected electrophysiological measures of E/I imbalance in pyramidal neurons (29). Similarly, IPA restored deficient inhibitory synaptic transmission in the hippocampus (13). At the synaptic level, metabolites influence pruning (14), gene expression related to synaptic proteins (37), and ER stress (38).

It is crucial to view these mechanisms not as isolated linear pathways but as components of an integrated system. The gut microbiota’s metabolic output is context-dependent, influenced by host diet, inflammation, and genetics. For example, during active ulcerative colitis, microbial metabolism shifts to produce immunomodulatory tryptophan metabolites or metabolize drugs, directly linking microbial metabolic capacity to disease activity and treatment response (43). Similarly, the baseline gut microbial metabolic profile, particularly involving bile acid and amino acid metabolism, can predict the success of clinical interventions like peanut oral immunotherapy, indicating a profound role for the microbiome in shaping host immunological tone (18). Furthermore, these interactions exhibit significant individuality. Early-life gut inflammation induces sex-dependent changes in the microbiome-endocrine-brain axis, affecting circulating androgens and leading to sex-specific alterations in microglial morphology and behavior (44). Host genetic polymorphisms in receptors like AHR further personalize the response to microbial metabolites (40). Therefore, the mechanistic impact of any given metabolite is filtered through a lens of developmental timing, host sex, genetic background, and concurrent immunological state, which collectively determine the final neurological and behavioral outcome. Notably, how these diverse metabolites interact synergistically or antagonistically at the BBB or within neural circuits remains largely unknown and represents a critical direction for future investigation. Furthermore, the precise molecular mechanisms by which specific metabolites interact with sex hormone signaling pathways remain unknown. These represent genuine knowledge gaps in the current literature, and we highlight them as priorities for future investigation.

## 5 The early-life window: perinatal and childhood influences on the metabolite-producing microbiome

**Figure 3:**
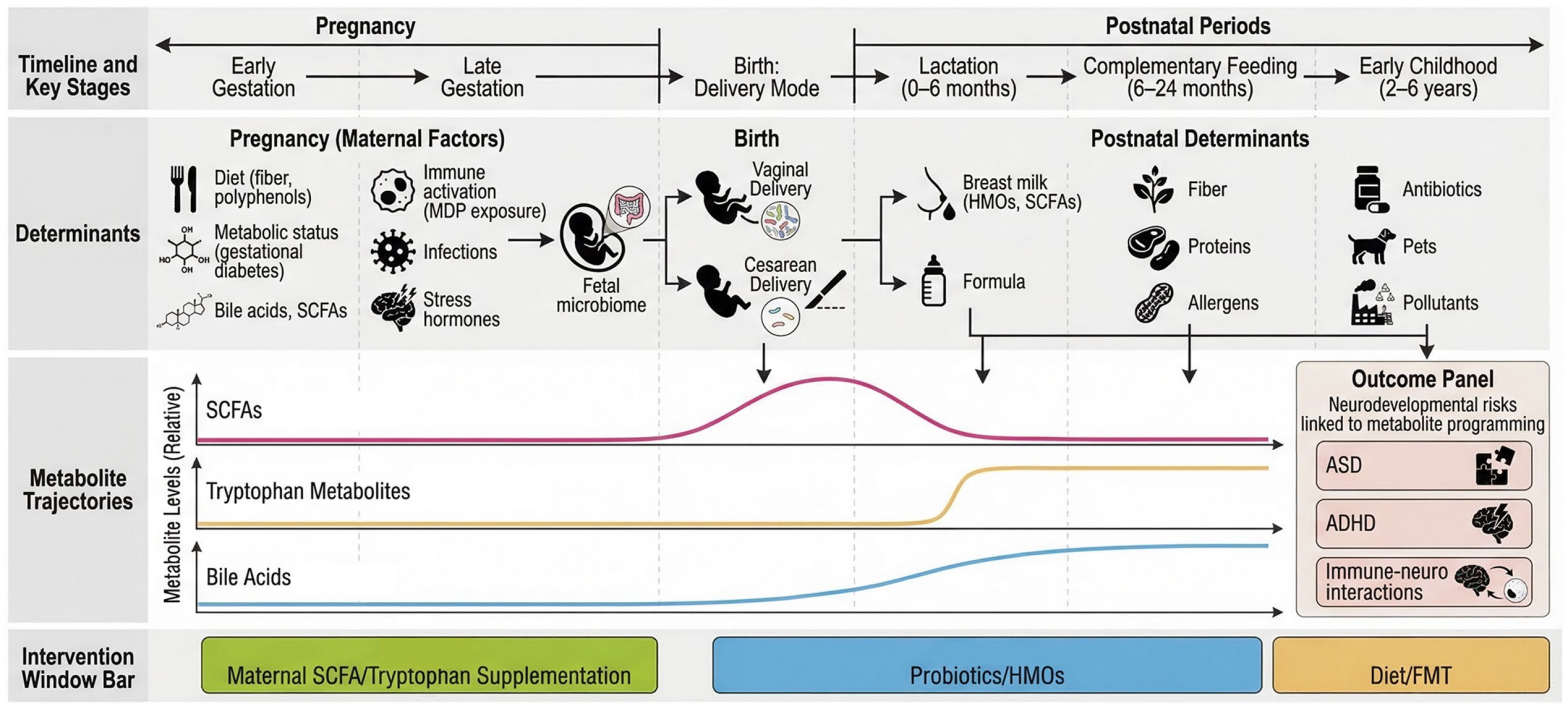
The first 1,000 days: shaping the metabolite-producing microbiome. Temporal dynamics of microbial metabolite programming across the perinatal and early childhood window, with associated modulatory factors and intervention opportunities. The timeline spans from preconception through early gestation, late gestation, birth, lactation (0–6 months), complementary feeding (6–24 months), and early childhood (2–6 years). (Upper panel) Key determinants shaping the developing microbiome and its metabolic output at each stage. During pregnancy, maternal factors including diet, metabolic status (e.g., gestational diabetes), bile acid profiles, and immune activation [e.g., exposure to microbial components like muramyl dipeptide (MDP)] influence fetal metabolic exposure. The mode of delivery (vaginal versus Cesarean section) determines initial colonization patterns. Postnatally, feeding practices—breast milk [containing human milk oligosaccharides (HMOs) and intrinsic SCFAs] versus formula—shape microbial assembly and metabolite availability. Complementary feeding introduces dietary fibers and proteins that diversify microbial metabolism. Environmental exposures, including antibiotics, pet ownership, and pollutants [per- and polyfluoroalkyl substances (PFAS), microplastics], continually modulate the ecosystem. (Middle panel) Trajectories of key metabolite classes across development. SCFAs peak during breastfeeding and complementary feeding; tryptophan metabolism undergoes a critical transition with the introduction of solid foods; bile acid profiles mature toward an adult-like configuration by age 2–3 years. (Lower panel) Neurodevelopmental outcomes linked to early-life metabolic programming, including risk for autism spectrum disorder (ASD), attention-deficit/hyperactivity disorder (ADHD), and immune-brain interactions. (Bottom bar) Windows of opportunity for preventive and therapeutic interventions, including maternal metabolite supplementation during pregnancy, probiotic and HMO administration in infancy, and dietary or microbial interventions [e.g., fecal microbiota transplantation (FMT)] in early childhood. Color intensity represents the relative magnitude or activity of each factor over time. MDP, muramyl dipeptide; HMO, human milk oligosaccharide; SCFAs, short-chain fatty acids; PFAS, per- and polyfluoroalkyl substances; FMT, fecal microbiota transplantation; ASD, autism spectrum disorder; ADHD, attention-deficit/hyperactivity disorder. Created with BioRender.com.

The profound context-dependency of host-microbiome interactions is perhaps most critically exemplified during the perinatal period and early childhood, a phase characterized by unparalleled plasticity. This developmental window represents a foundational period for the assembly of the gut ecosystem and its metabolic programming, setting trajectories that can influence neurodevelopment and long-term health (33). Figure 3 illustrates the temporal dynamics of this critical window, highlighting the key modulatory factors across the first 1,000 days and the corresponding windows of opportunity for preventive interventions. The composition and function of the early-life microbiome, and consequently its metabolic output, are shaped by a cascade of sequential and interacting factors originating from the mother, the mode of birth, postnatal nutrition, and early-life exposures. Disturbances during this sensitive period can lead to dysbiosis and an altered microbial metabolome, creating a substrate that may predispose to immune dysfunction and aberrant neurodevelopment (44, 45).

Maternal factors constitute the primary environmental imprint on the offspring’s microbial and metabolic landscape. Maternal diet during gestation and lactation directly influences her own gut microbiota and the metabolites available to the fetus and neonate. For instance, a maternal Mediterranean-like diet rich in fiber and vegetable protein enhanced maternal intestinal health and mucosal immunity, and increased cecal SCFA production, compared to a more Western-like diet (46). Conversely, maternal over nutrition, such as a high-fat diet, can program adverse metabolic outcomes in offspring through epigenetic mechanisms, including the suppression of key hepatic enzymes like cholesterol 7α-hydroxylase (CYP7A1) involved in cholesterol and bile acid metabolism (47). Beyond diet, the maternal metabolic and inflammatory state has significant consequences. Gestational diabetes mellitus (GDM) alters the maternal gut microbiota, reducing SCFA production, which in a mouse model impaired GPR43 signaling in the fetus and led to congenital anomalies of the kidney and urinary tract (8). Similarly, maternal intrahepatic cholestasis of pregnancy (ICP), particularly early-onset ICP, is associated with a significantly increased risk of neurodevelopmental conditions in offspring, implicating disrupted maternal bile acid metabolism as a potential contributing factor (48). The maternal microbiome can influence stem cell function in offspring. Researchers transplanted different maternal microbiomes into germ-free mice, resulting in changes to the proliferation and differentiation of neuronal and intestinal stem cells in the offspring. These effects were mediated by circulating microbial metabolites like SCFAs and dependent on mTOR signaling (49). Even without overt infection, maternal exposure to microbial components like MDP during late pregnancy can lead to sex-specific alterations in neurodevelopment and social behavior in juvenile offspring, highlighting a direct pathway for maternal microbiome-derived signals to influence the fetal brain (37). Maternal psychological stress and vulnerability are also reflected in altered tryptophan metabolism, with higher maternal vulnerability scores associated with lower plasma tryptophan concentrations, potentially affecting the substrate available for microbial conversion into neuroactive indoles (50).

The initial colonization event at birth and subsequent postnatal feeding practices are decisive forces that sculpt the nascent microbiome. The mode of delivery determines the source of pioneer bacteria, while breastfeeding provides both live microbes (including key *Bifidobacterium* species) and prebiotic human milk oligosaccharides (HMOs) that selectively nourish a specific, health-associated microbiota (51). Breastfeeding is the strongest factor associated with infant stool consistency, a proxy for gut transit time, which in turn shapes the fecal metabolome, including SCFA and bile acid amidate profiles (52). The protective effects of breastfeeding may be partly mediated by microbial metabolites; for example, specific SCFAs like formic, acetic, and caproic acid in infant plasma at 4 months are negatively associated with subsequent sensitization and atopic disease (5). Notably, butyric and caproic acid are enriched approximately 100-fold in human breast milk compared to maternal plasma, suggesting potential active transport to meet infant needs (5). The importance of early microbial metabolites is further underscored by the finding that the temporal pattern of *Bifidobacterium* colonization in the first week of life, and its associated metabolite ILA measured in neonatal dried blood spots, is linked to the risk of developing ADHD at age 10 (33).

The immature early-life ecosystem is highly vulnerable to perturbation. Antibiotic exposure, a common intervention, can disrupt this developing community. While one study in healthy term infants found antibiotic-associated microbiome changes to be minimal and transient compared to the overwhelming effects of breastfeeding and age (53), other contexts reveal more significant impacts. Early-life antibiotic challenge in mice reduced SCFA content and diversity, effects that could be revived by supplementation with *Bifidobacterium longum* subsp. *infantis* and the HMO 2′-fucosyllactose (2′-FL) (54). Prolonged antibiotic courses in pediatric patients can lead to complications like neutropenia, which is associated with depletion of beneficial *Lachnospiraceae* and a reduction in their associated metabolites, including those in the urea cycle (55). Early-life illnesses also reshape the metabolome; for example, infants with cow’s milk protein allergy exhibit gut dysbiosis and altered SCFA profiles (56, 57). Furthermore, critical illnesses requiring interventions like cardiopulmonary bypass (CPB) surgery in neonates induce significant postoperative changes in the profile of indole metabolites, with specific metabolites like indole-3-carboxylic acid associated with better clinical outcomes, while others like ILA were linked to worse outcomes (58).

Nutritional status and pharmacological exposures present additional modifiers. Protein malnutrition in juvenile mice facilitates persistent intestinal colonization by antibiotic-resistant *Klebsiella pneumoniae*, a susceptibility linked to a reduction in inhibitory secondary bile acids (59). Early exposure to pharmaceuticals such as the antipsychotic risperidone induces gut dysbiosis, damages the intestinal barrier, and alters brain levels of microbial metabolites, including reducing the neuroprotective bile acid TUDCA and increasing the neurotoxic tyrosine metabolite p-cresol, collectively contributing to cognitive impairment (38). Environmental toxicants like PFAS can also intersect with this axis; prenatal PFHxS exposure was associated with altered gut microbiota diversity, a decrease in SCFA-producing genera like *Ruminococcus*, and increased neurobehavioral problems in childhood, with microbiota changes partially mediating this association (36).

The confluence of these early-life factors ultimately determines the functional output of the gut microbiome—its metabolome—which interfaces with host physiology. The trajectory of MCBAs in early life is distinct and associated with immune development; altered levels of specific MCBAs are linked to the development of islet autoimmunity and modulate immune cell function (60). Early-life gut inflammation, as modeled in juvenile mice, induces sex-dependent shifts in the microbiome-endocrine-brain axis, altering circulating androgens and leading to chronic changes in microglial morphology (44). Notably, the associations between microbial metabolic potential and later neurodevelopment are evident in human cohorts. In a large U. S. multicenter study, higher abundance of microbial genes encoding SCFA synthesis pathways and higher fecal butyrate concentrations in infancy were prospectively associated with higher scores indicative of ASD-related social impairments (61).

The plasticity of this early window also presents a unique opportunity for intervention. Maternal supplementation with microbial tryptophan metabolites like indole or indole-3-acetic acid during gestation and lactation protected offspring from diet-induced metabolic liver disease in adulthood by sustaining AHR activation and altering hepatic ceramide profiles (62). Postnatal supplementation with 2’-FL improved ASD-like behaviors in a mouse model, an effect mediated by the gut microbiota and associated with increased beneficial bacteria and elevated brain bile acid signaling (30). These preclinical findings, alongside observations that microbial indole metabolites can inhibit rotavirus infection in infants (63), underscore the potential of targeting the early-life microbial metabolome for prevention and therapy. Thus, the early-life window is not merely a period of vulnerability but also a pivotal phase for shaping a resilient microbial metabolic network that supports healthy neurodevelopment.

## 6 Toward translation: dietary, probiotic, and microbial intervention strategies

Building on the notion that the early-life microbial metabolome is a malleable and pivotal network, a growing body of preclinical and clinical research is actively exploring targeted strategies to modulate this system for neurodevelopmental benefit (6, 64). These interventions, ranging from dietary modifications and microbial supplementation to more direct microbial manipulations, aim to correct dysbiosis and restore a favorable profile of neuroactive microbial metabolites, thereby offering potential avenues for managing NDDs.

Probiotic administration, involving live beneficial bacteria, represents a direct approach to modify the gut microbial community. In rodent models of ASD, specific probiotic strains have demonstrated efficacy in ameliorating behavioral deficits. For instance, supplementation with *Lactobacillus reuteri* or *Lactobacillus rhamnosus* GG in a maternal immune activation model improved social behavior in offspring, an effect linked to enhanced gut barrier integrity, modulation of SCFA profiles (increased butyrate, decreased propionate), and attenuation of systemic inflammation and HPA axis over activation (26). Similarly, *Bifidobacterium adolescentis* DM8504 alleviated autistic-like behaviors in a valproic acid-induced model, concomitant with the restoration of fecal SCFA levels and enrichment of SCFA-producing bacteria like *Faecalibacterium* and *Lachnospiraceae_NK4A136_group* (27). These findings underscore that the beneficial effects of specific probiotics may be mediated, at least in part, through their capacity to reshape the microbial metabolic output. The concept is extended by synbiotics, which combine probiotics with prebiotic substrates to selectively promote their growth. An encapsulated synbiotic containing *Limosilactobacillus fermentum* K73, when tested *in vitro* with fecal samples from children with ASD, induced favorable microbial shifts and increased the production of butyric acid while decreasing propionic acid (65). In a subsequent pilot study using gnotobiotic mice colonized with microbiota from children with ASD, supplementation with this synbiotic induced beneficial microbial changes and associated behavioral improvements (66). In a randomized controlled trial involving individuals with ADHD, a multi-strain synbiotic (Synbiotic 2000) not only reduced psychiatric symptoms but also induced specific changes in the fecal microbiome’s taxonomy and function, alongside increasing plasma SCFA levels toward those observed in controls (32).

Dietary interventions offer a broader, substrate-based strategy to influence the gut microbial ecosystem. Specific nutrients can directly serve as precursors for microbial metabolites or shape the community of metabolite-producing bacteria. Dietary supplementation with L-tyrosine in an ASD mouse model significantly mitigated autistic-like behaviors, an effect strongly connected to amended gut microbial composition and function, as transplantation of the L-tyrosine-modulated microbiota recapitulated the behavioral benefits (28). Human milk oligosaccharides, such as 2′-fucosyllactose (2′-FL), have garnered interest for their prebiotic properties. Postnatal supplementation with 2′-FL improved ASD-like behaviors in a mouse model, effects mediated by the gut microbiota and associated with increased beneficial bacteria (e.g., *Akkermansia*, *Bifidobacterium*) and elevated brain bile acid signaling (30). Furthermore, early-life supplementation with *Bifidobacterium longum* ssp. *infantis* combined with 2′-FL mitigated antibiotic-induced gut dysbiosis and supported intestinal and immune development in mice (54). At a broader dietary pattern level, a maternal diet during gestation and lactation rich in fiber and vegetable protein (akin to a Mediterranean diet) enhanced maternal mucosal immunity and cecal SCFA production, likely mediated by beneficial modifications in the cecal microbiota (46). Conversely, exposure to environmental contaminants like certain PFAS alternatives has been associated with reduced gut microbial diversity, altered metabolite profiles, and decreased SCFA levels in children, highlighting the detrimental impact of some modern environmental factors on the metabolite-producing microbiome (67).

FMT constitutes a more aggressive intervention aimed at rapidly overhauling the recipient’s gut microbial community. In a propionic acid-induced mouse model of ASD, targeted FMT from healthy human donors selected for high *Lactobacillus* abundance ameliorated behavioral deficits, restored gut microbiota diversity, reduced propionic acid levels in the brain, and normalized the excitatory/inhibitory balance in the prefrontal cortex (29). In pediatric ulcerative colitis (UC), FMT induced significant and sustained increases in gut microbial diversity over 48 weeks and was associated with specific microbial signatures linked to clinical improvement (68). These studies illustrate the potential of FMT to induce durable microbial and metabolic changes, although the high placebo response rate in some trials underscores the complexity of this intervention (68). The causal role of the gut microbiome is further reinforced by FMT experiments in other models; for example, transplantation of microbiota from risperidone-treated mice to naïve recipients transferred cognitive impairment, directly implicating drug-induced dysbiosis in the side effect (38).

Beyond the introduction of live microbes, interventions can also target microbial metabolites or the pathways they influence. Butyrate supplementation has shown promise in various models. It rescued social deficits induced by embryonic exposure to the pesticide chlorpyrifos in zebrafish, an effect linked to the inhibition of class I histone deacetylases like HDAC1 (10). In a TS rat model, the therapeutic effect of Jing An decoction, a traditional Chinese medicine formulation, was mediated by enriching butyrate-producing bacteria and elevating butyrate levels, which subsequently alleviated neuroinflammation by inhibiting the TLR4/HDAC3/NF-κB pathway in microglia (9). Similarly, in a food allergy model, butyrate alleviated allergic reactions by improving intestinal barrier integrity and suppressing oxidative stress-mediated Notch signaling (39). Other microbial metabolites are also being explored. Maternal supplementation with the tryptophan derivatives indole or indole-3-acetic acid during gestation and lactation conferred long-term protection against diet-induced metabolic liver disease in offspring by sustaining AHR activation (62). Furthermore, certain traditional herbal formulations exert their effects by remodeling the microbial metabolome. For example, Xiaoer Huanglong Pellets improved ADHD-like behaviors in rats by restoring gut microbial homeostasis and correcting imbalances in amino acid metabolism and SCFA production (34). Xiaoer Qixing Cha alleviated constipation in mice by restoring gut microbiota and SCFA levels, particularly butyrate and isobutyrate (69).

A successful precedent for metabolite-targeted therapy comes from hepatology, where understanding bile acid enterohepatic circulation led to the development of ileal bile acid transporter (IBAT) inhibitors, now approved for pediatric cholestatic pruritus (70–74). This paradigm illustrates how mechanistic insights into host–microbe metabolic dialog can yield transformative therapies—a concept directly relevant to the neurodevelopmental field.

Collectively, these diverse strategies—probiotics, synbiotics, dietary components, FMT, and metabolite-targeted therapies—converge on the principle of modifying the gut microbial ecosystem to favorably influence its metabolic output. While preclinical evidence is compelling, clinical application requires careful consideration. The efficacy appears to be strain-specific, condition-dependent, and likely influenced by the individual’s baseline microbiota. Furthermore, the safety and long-term consequences of interventions like FMT or early-life probiotic supplementation in children require rigorous evaluation (75). The integration of microbial and metabolomic profiling holds promise for personalizing these interventions, moving toward a future where modulation of the microbiota-gut-brain axis can be tailored to correct specific metabolic deficiencies implicated in NDDs. Table 2 provides a comprehensive summary of these intervention strategies, detailing their target metabolites, observed metabolic effects, model systems, behavioral outcomes, and evidence levels, thereby offering a practical reference for evaluating the translational potential of microbiome-based therapies in NDDs.

**Table 2:** Intervention strategies targeting the microbiota-metabolite-brain axis in neurodevelopmental disorders.

Intervention category	Specific intervention	Target metabolite(s)/pathway	Observed metabolic effects	Model system	Behavioral/clinical outcomes	Developmental stage	Evidence level	References
Probiotics	*Lactobacillus reuteri*	SCFA profile	↑ Fecal butyrate; ↓ propionate	MIA mouse model (ASD)	Improved social behavior; ↓ systemic inflammation; enhanced gut barrier	Postnatal	Preclinical	(26)
	*Lactobacillus rhamnosus* GG	SCFA profile	↑ Fecal butyrate; ↓ propionate	MIA mouse model (ASD)	Improved social behavior; ↓ corticosterone; enhanced gut barrier	Postnatal	Preclinical	(26)
	*Bifidobacterium adolescentis* DM8504	SCFA profile	Restored fecal SCFA levels; ↑ SCFA-producing taxa	VPA rat model (ASD)	Alleviated autistic-like behaviors	Postnatal	Preclinical	(27)
Synbiotics	*Limosilactobacillus fermentum* K73 + prebiotic	SCFA profile	↑ Butyric acid; did not increase propionic acid	*In vitro* (ASD child fecal culture)	Favorable microbial community shifts	Pediatric	*In vitro*	(65)
	*L. fermentum* K73 synbiotic	SCFA profile and microbial function	↑ Key SCFAs; promoted beneficial genera	Gnotobiotic mice colonized with ASD microbiota	Improved social behavior	Postnatal	Preclinical	(66)
	Synbiotic 2000 (*Pediococcus pentosaceus*, etc.)	SCFA profile	↑ Plasma SCFAs toward control levels	RCT in children with ADHD	↓ Psychiatric symptoms; ↑ *Prevotella*	Pediatric	Clinical (RCT)	(32)
Dietary interventions	L-Tyrosine supplementation	Catecholamine PRECURSOR availability	Remodeled gut microbiota; ↑ tyrosine-derived metabolites	BTBR mouse model (ASD)	Mitigated autistic-like behaviors; effects transferable via FMT	Postnatal	Preclinical	(28)
	2′-fucosyllactose (2′-FL)	Bile acid metabolism and microbial ecology	↑ Brain bile acid levels (TCA, TCDCA); ↑ *Akkermansia*, *Bifidobacterium*	MIA mouse offspring (ASD)	Improved ASD-like behaviors	Postnatal (growth period)	Preclinical	(30)
	2′-FL + *B. longum* ssp. *infantis*	Microbial ecology and immune development	Mitigated antibiotic-induced dysbiosis	Antibiotic-exposed neonatal mice	Supported intestinal and immune development	Early-life	Preclinical	(54)
Fecal microbiota transplantation	FMT from healthy donors (high *Lactobacillus*)	SCFA profile	↓ Brain propionic acid; restored microbial diversity	Propionic acid-induced ASD mouse model	Ameliorated behavioral deficits; normalized PFC E/I balance	Postnatal	Preclinical	(29)
	FMT from risperidone-treated Mice	Bile acids and phenolic compounds	↓ Beneficial bile acids (e.g., TUDCA); ↑ p-Cresol	Naïve recipient mice	Transferred cognitive impairment (novel object recognition deficit)	Postnatal	Preclinical	(38)
Prevention and early intervention	Perinatal *L. rhamnosus* GG	Early microbial colonization	Modulated infant gut microbiota composition	Human RCT (mothers-infants)	Reduced cumulative risk of ASD/ADHD diagnosis by age 13	Perinatal to infancy	Clinical (RCT, preventive)	(85)
Traditional Chinese medicine	Jing an decoction	Butyrate production	↑ Butyrate (colon and striatum); ↑ butyrate-producing *Lachnospiraceae*	TS rat model	Alleviated tic-like behaviors; ↓ neuroinflammation; ↑ M2 microglia	Postnatal	Preclinical	(9)
	Xiaoer Huanglong Pellets	Amino acid and SCFA metabolism	Corrected amino acid dysregulation; restored SCFA production	ADHD rat model	Improved ADHD-like behaviors; repaired gut and BBB integrity	Postnatal	Preclinical	(34)
Direct metabolite supplementation	Butyrate (sodium butyrate)	HDAC inhibition	HDAC3 inhibition; ↓ TLR4/NF-κB signaling	TS rat model (mechanistic study)	Underlies anti-neuroinflammatory effects of Jing An Decoction	Postnatal	Preclinical (mechanistic)	(9)
	Indole-3-propionic acid (IPA)	Neuronal signaling (ERK1)	Activated ERK1 pathway in hippocampus	16p11.2 microdeletion mouse model (ASD)	Rescued social and cognitive deficits; restored inhibitory transmission	Postnatal	Preclinical	(13)
Environmental exposure mitigation	Butyrate (sodium butyrate)	HDAC inhibition	Reversed CPF-induced histone hypoacetylation and neuronal gene suppression	Zebrafish (Chlorpyrifos-induced social deficit model)	Rescued social interaction deficits	Embryonic/postnatal	Preclinical	(10)
Pharmacotherapy (mechanistically relevant)	Odevixibat (IBAT Inhibitor)	Bile acid pool	↓ Serum bile acids	Patients with PFIC, alagille syndrome (pediatric)	↓ Pruritus; ↓ serum bile acids; approved for cholestatic pruritus	Pediatric	Clinical (approved for non-NDD)	(70–74)
	Maralixibat (IBAT inhibitor)	Bile acid pool	↓ Serum bile acids	Patients with PFIC, alagille syndrome (Pediatric)	↓ Pruritus; improved quality of life; approved for cholestatic pruritus	Pediatric	Clinical (approved for non-NDD)	(72–76)

SCFA, short-chain fatty acid; MIA, maternal immune activation; VPA, valproic acid; ASD, autism spectrum disorder; ADHD, attention-deficit/hyperactivity disorder; TS, Tourette syndrome; PFIC, progressive familial intrahepatic cholestasis; RCT, randomized controlled trial; FMT, fecal microbiota transplantation; TUDCA, tauroursodeoxycholic acid; PFC, prefrontal cortex; E/I, excitatory/inhibitory; HDAC, histone deacetylase; TLR4, Toll-like receptor 4; NF-κB, nuclear factor kappa-light-chain-enhancer of activated B cells; BBB, blood–brain barrier; ERK, extracellular signal-regulated kinase; IBAT, ileal bile acid transporter; CPF, chlorpyrifos; NDD, neurodevelopmental disorder; Refs, references.

## 7 Conclusions and future perspectives: from association to causation and personalized approaches

The burgeoning field of research on the microbiota-gut-brain axis has fundamentally reshaped our understanding of NDDs. A compelling body of evidence, extensively reviewed in the preceding sections, now robustly associates specific alterations in gut microbial communities and, crucially, their metabolic output with conditions such as ASD and ADHD (2, 64, 76). The recurring themes of reduced SCFA production, disrupted tryptophan metabolism, and broader inflammatory and metabolic dysregulation across multiple NDDs suggest that microbial metabolites are not merely bystanders but potentially active contributors to the pathophysiology (77, 78). Preclinical models have provided mechanistic plausibility, demonstrating that these gut-derived molecules can influence neurodevelopment, immune tone, and behavior via endocrine, neural, and immune pathways (1, 3). This convergence of associative human data from observational studies and mechanistic animal studies has fueled enthusiasm for microbiome-targeted interventions, ranging from dietary modifications and probiotics to more drastic approaches like FMT (2, 76).

However, the current state of the field remains largely in the realm of association. A critical limitation across many studies is their cross-sectional or retrospective design, which precludes the establishment of temporal sequence and definitive causality (79, 80). While dysbiosis and metabolite alterations are consistently reported in affected children, it remains challenging to disentangle whether these changes are primary drivers, secondary consequences of altered diet or behavior common in NDDs, or epiphenomena of shared genetic or environmental risk factors (4, 78). The high degree of individual heterogeneity in gut microbiota composition further complicates the identification of universal microbial or metabolic signatures for specific disorders (1, 2). Future research must prioritize large-scale, longitudinal birth cohort studies that profile the developing microbiome and metabolome alongside detailed neurodevelopmental assessments. Such studies are essential to determine if specific microbial-metabolic trajectories precede and predict the onset of symptoms, thereby strengthening causal inference (4, 81). Integrating multi-omics data—metagenomics, metabolomics, proteomics, and host genomics—will be paramount in moving beyond taxonomic descriptions to a functional understanding of host–microbe interactions in neurodevelopment (3, 82).

The pursuit of causality must also extend to a more nuanced consideration of environmental and evolutionary contexts. The hypothesis that certain neurodivergent phenotypes, like some presentations of ADHD, may represent an evolutionary mismatch—where a gut-brain axis tuned for pathogen-rich ancestral environments malfunctions in modern sanitized settings—offers a provocative framework for understanding individual susceptibility (64). This perspective aligns with observations linking early-life factors known to shape the microbiome (e.g., birth mode, antibiotic use, pet exposure) to NDD risk (24). Furthermore, emerging evidence on environmental pollutants like microplastics altering the gut microbiome and its metabolic capacity (e.g., reducing butyrate) introduces another layer of complexity, suggesting that the modern exposome may be interactively eroding the resilience of the microbiota-gut-brain axis (80). Future mechanistic studies should employ advanced models, such as organ-on-chip systems that integrate human cells and microbial communities, to dissect these complex interactions under controlled yet physiologically relevant conditions (3).

On the translational front, the path toward effective and personalized microbiome-based therapies is fraught with challenges but holds significant promise. Current intervention studies, particularly with probiotics, often yield mixed results, underscoring the likely strain-specific and individual nature of responses (23, 78). The future of microbial therapeutics lies in precision approaches. This will require the development of diagnostic frameworks that combine microbial and metabolomic profiling to identify specific, functionally relevant deficiencies in an individual—be it a lack of butyrate-producing bacteria, an overabundance of pro-inflammatory pathobionts, or a skewed bile acid pool (2, 76). Interventions could then be tailored, whether through next-generation probiotics (e.g., *Akkermansia muciniphila* or engineered strains), prebiotics designed to nourish deficient taxa, targeted postbiotics (specific microbial metabolites), or even CRISPR-based microbial editing (3, 23). The concept of treating NDDs may also expand to address frequent co-morbid conditions, such as dermatological or gastrointestinal issues, through a unified gut-brain-skin axis approach, potentially improving overall quality of life with integrated strategies (77).

Notably, insights from pediatric research in other organ systems highlight both the potential and the caution needed. In pediatric metabolic liver disease, gut-derived metabolites like SCFAs and bile acids are recognized as key pathogenic players and therapeutic targets (81, 83). The successful development of ileal bile acid transporter (IBAT) inhibitors for cholestatic pruritus, pioneered in children with rare liver diseases, demonstrates how targeting a specific gut-liver axis pathway can transform clinical management (73, 74). This serves as a powerful precedent for the NDD field, illustrating that a deep mechanistic understanding of host-microbe metabolic dialog can yield transformative therapies. Conversely, research in areas like childhood asthma and Kawasaki disease reveals the difficulties in moving from associative microbial signatures to proven causal mechanisms and effective interventions, mirroring the challenges in NDDs (79, 84). These parallel fields emphasize the necessity for rigorous, well-controlled clinical trials that move beyond small pilot studies to establish efficacy and safety definitively (4, 23).

**Figure 4:**
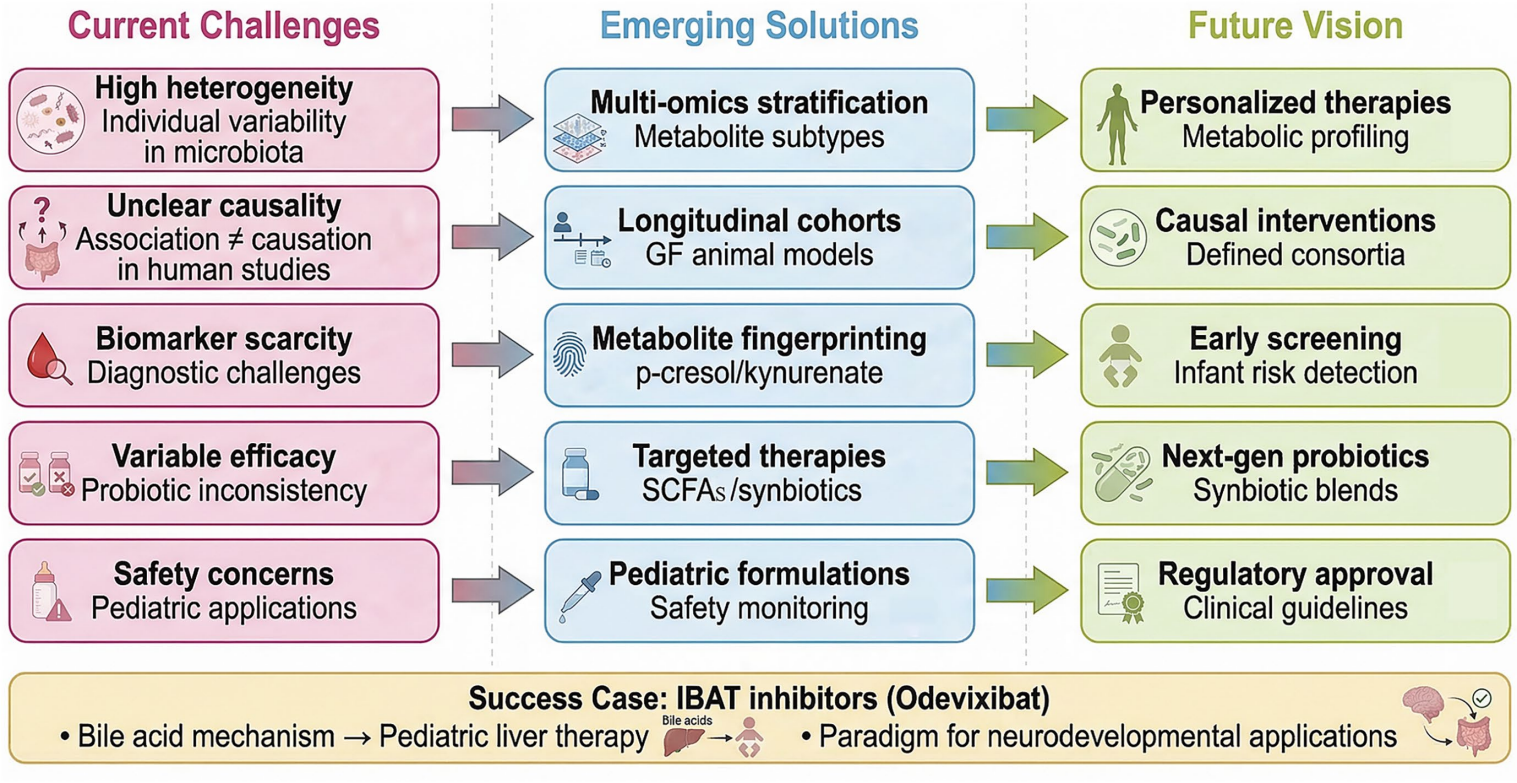
Translating microbial metabolite science into clinical practice: a roadmap. Schematic illustrating the current challenges, emerging solutions, and future vision for translating gut microbial metabolite research into clinical applications for neurodevelopmental disorders. (Left panel) Key barriers to translation include high inter-individual heterogeneity in gut microbiota composition, unresolved causality versus association in human studies, lack of validated diagnostic biomarkers, inconsistent efficacy of probiotic interventions, and safety concerns specific to pediatric populations. (Center panel) Corresponding solutions under development: multi-omics stratification to define metabolite-based subtypes; longitudinal birth cohorts combined with germ-free animal models to establish causality; metabolite fingerprinting (e.g., p-cresol, kynurenate) for early screening; targeted interventions including SCFA supplementation and next-generation synbiotics; and pediatric-specific formulations with long-term safety monitoring. (Right panel) The envisioned future of personalized microbiome medicine: individualized microbial therapeutics based on metabolic profiling, causal interventions using defined microbial consortia or metabolites, early-life screening tools for at-risk infants, and regulatory-approved therapies integrated into clinical guidelines. (Bottom panel) A precedent-setting success story: the development of ileal bile acid transporter (IBAT) inhibitors (odevixibat, maralixibat) for pediatric cholestatic diseases. This pathway—from mechanistic understanding of bile acid signaling to transformative therapy in children—provides a paradigm for the neurodevelopmental field. GF, germ-free; SCFAs, short-chain fatty acid; IBAT, ileal bile acid transporter. Created with BioRender.com.

In conclusion, the role of microbial metabolites in neurodevelopment represents a paradigm shift, opening new avenues for understanding, preventing, and treating complex disorders like ASD and ADHD. The evidence for association is strong and mechanistically supported. The imperative now is to rigorously test causality, embrace the complexity of individual and environmental interactions, and develop a new generation of targeted, personalized microbial therapeutics. Achieving this goal will require a concerted, interdisciplinary effort integrating microbiology, neuroscience, metabolomics, and clinical pediatrics. By doing so, the field can evolve from mapping correlations to engineering solutions, ultimately harnessing the power of our microbial partners to support healthier brain development. Furthermore, beyond the few examples discussed in this review (e.g., risperidone), how a broader range of standard NDD medications interact bidirectionally with the gut-brain-metabolite axis remains largely unexplored. Whether these drugs interfere with microbiome-based therapies or whether the microbial metabolome alters drug metabolism (pharmacometabolomics) represents a critical gap that requires urgent investigation. Figure 4 synthesizes the translational roadmap for the field, outlining current challenges, emerging solutions, and a future vision for personalized microbiome-based therapies, using the successful development of IBAT inhibitors in pediatric cholestatic diseases as a precedent-setting paradigm.
